# Eprobe-mediated screening system for somatic mutations in the *KRAS* locus

**DOI:** 10.3892/or.2015.3883

**Published:** 2015-03-30

**Authors:** JUN ATSUMI, TAKESHI HANAMI, YASUAKI ENOKIDA, HIROOMI OGAWA, DIANE DELOBEL, YASUMASA MITANI, YASUMASA KIMURA, TAKAHIRO SOMA, MICHIHIRA TAGAMI, YOSHIAKI TAKASE, TATSUO ICHIHARA, IZUMI TAKEYOSHI, KENGO USUI, YOSHIHIDE HAYASHIZAKI, KIMIHIRO SHIMIZU

**Affiliations:** 1 Departments of Thoracic and Visceral Organ Surgery, Gunma University Graduate School of Medicine, Maebashi, Japan; 2 Division of Genomic Technologies, RIKEN Center for Life Science Technologies, Yokohama, Kanagawa, Japan; 3 K.K. DNAFORM, Yokohama, Kanagawa, Japan; 4 RIKEN Preventive Medicine and Diagnosis Innovation Program, Yokohama, Kanagawa, Japan

**Keywords:** Eprobe, competing effect, Kristen rat sarcoma viral oncogene homolog, colorectal cancer

## Abstract

Activating mutations in the Kirsten rat sarcoma viral oncogene homolog (*KRAS*) loci are largely predictive of resistance to epidermal growth factor receptor (EGFR) therapy in colorectal cancer (CRC). A highly sensitive detection system for the *KRAS* gene mutations is urgently needed; however, conventional methods have issues with feasibility and cost performance. Here, we describe a novel detection system using a fluorescence ‘Eprobe’ capable of detecting low level *KRAS* gene mutations, via real-time PCR, with high sensitivity and simple usability. We designed our Eprobes to be complementary to wild-type (WT) *KRAS* or to the commonly mutated codons 12 and 13. The WT Eprobe binds strongly to the WT DNA template and suppresses amplification by blocking annealing of the primer during PCR. Eprobe-PCR with WT Eprobe shows high sensitivity (0.05–0.1% of plasmid DNA, 1% of genomic DNA) for the *KRAS* mutation by enrichment of the mutant type (MT) amplicon. Assay performance was compared to Sanger sequencing using 92 CRC samples. Discrepancies were analyzed by mutation genotyping via Eprobe-PCR with full match Eprobes for 7 prevalent mutations and the next generation sequencing (NGS). Significantly, the Eprobe system had a higher sensitivity for detecting *KRAS* mutations in CRC patient samples; these mutations could not be identified by Sanger sequencing. Thus, the Eprobe approach provides for highly sensitive and convenient mutation detection and should be useful for diagnostic applications.

## Introduction

The Kirsten rat sarcoma viral oncogene homolog (KRAS) protein is an important signaling mediator in the epidermal growth factor receptor (EGFR) pathway, which regulates cell growth and is commonly targeted in cancer therapy (1,2). Mutations in codon 12 and 13 of the *KRAS* gene are found in ~30% of colorectal cancer (CRC) cases and are associated with an increased risk of recurrence and mortality (3). Various preclinical and clinical studies have revealed that the presence of KRAS activating mutations in CRC correlates with resistance to EGFR monoclonal antibodies (EGFR mAb), such as cetuximab (Erbitax) and panitumumab (Vectibix) (4–8). As such, the US Food and Drug Administration (FDA) has recommended that EGFR mAbs should not be given to patients with tumors harboring KRAS mutations in codon 12 or 13 (6). This requires the *KRAS* mutation status to be assessed prior to treatment, yet clinicians currently lack the ability to detect these mutations with a high sensitivity, high-throughput and simple method.

Sanger sequencing is the gold standard for detecting *KRAS* mutations; however, it is highly inefficient and has relatively low sensitivity. In response to increasing demands in the medical setting, various *KRAS* mutation detection assays have been developed and described in the literature and different providers offer commercial test kits (9,10). Real-time PCR based methods are the most cost-effective and require shorter working times than other methods. The Scorpion, Amplification Refractory Mutation System (ARMS), and TaqMelt systems are among the widely used real-time PCR-based *KRAS* mutation detection systems (11–13). While these are highly sensitive and reproducible methods, each has its advantages and disadvantages. The Scorpion and ARMS methods are based on mutation-matched primers, which amplify mutated loci more efficiently than primers with wild-type (WT) sequences or other mutations. Seven reactions are necessary to detect the mutation status and thus the amount of DNA needed is ~800 ng (12). Alternatively, the TaqMelt method is based on the melting curve analysis after PCR and is able to detect a total of 19 mutations in *KRAS* codons 12, 13 and 61, yet it requires sophisticated instruments and software. Hence, a more simple and cost-effective detection assay capable of producing high quality results may greatly benefit cancer diagnostics.

In addition to mutation sequencing at clinical laboratories, most of these assays use an amplification-dependent detection method sometimes combined with melting curve analysis of the amplicon. The use of high-throughput sequencing for detecting *KRAS* mutations has been described and some providers have started to offer dedicated cancer sequencing panels (14). While high-throughput sequencing has been used as a reference for testing the performance of new *KRAS* detection assays, this approach could be extended to wider applications. Assuming that PCR-based detection assays are able to achieve a mutation sensitivity greater than or equal to that of a Sanger sequencing-based approach, PCR-based detection assays should be sufficient to confirm mutations found by high-throughput sequencing.

In the present study, we aimed at developing a new PCR-based detection assay using Eprobe, or ‘Eprobe-mediated PCR’. Eprobe is a fluorescence probe enabling quantification analysis and melting curve analysis using real-time PCR machines (15–17). Eprobe binds complementary DNA with higher affinity than normal oligonucleotides by using cationic dye moieties (18) and thus leads to a competitive effect observed in primer annealing and extension. This characteristic enables specific sequence enrichment-similar to using peptide and locked nucleic acids (PNA and LNA, respectively) as a clamping probe (19–27), to allow for the detection of even miniscule amounts of mutated DNA. Notably, this method can detect somatic mutations with high accuracy in simple steps that employ commonly used laboratory equipment and a small amount of DNA, unlike other methods. Thus, Eprobe-mediated PCR enables efficient detection of mutation status and is a major advance in cancer diagnostics. Here, we demonstrate a novel method to detect *KRAS* mutations at codon 12 and 13 by Eprobe-mediated PCR with a higher sensitivity than conventional Sanger sequencing, which is more time and cost-effective than other technologies.

## Materials and methods

### Reagents and control DNA

DNA oligonucleotides were purchased from Sigma-Aldrich (Ishikari, Japan), and stored as 100 mM stock solutions in 10 mM Tris-HCl with 1 mM EDTA (pH 8.0). WT human genomic DNA was purchased from Promega (Tokyo, Japan). Heterozygote human genomic DNA with seven mutations (G12A, G12C, G12D, G12R, G12S, G12V and G13D) in the *KRAS* gene codon 12 and 13 were obtained from Horizon Diagnostics (Cambridge, UK). Eprobes (shown in Table I) were obtained from K.K. DNAFORM (Yokohama, Japan). Point mutations in codon 12 of the *KRAS* gene (GAT, GCT, GTT, AGT, TGT) were cloned into the plasmid pGEM-T (Promega) as previously described (26) and stored in glycerol at −80°C for long-term conservation. Point mutation of codon 13 (GAC) in the *KRAS* gene was prepared using the same protocol. The *KRAS* codon 12 (CGT) point mutation was prepared using the synthetic DNA ordered and inserted into a pUC57 plasmid (GenScript, Tokyo, Japan). The sequences of WT and all seven mutant clones were verified on ABI PRISM 3100 Avant (Applied Biosystems, Tokyo, Japan), diluted in TE buffer and stored at −20°C until use.

### Design of Eprobe and real-time PCR

Human *KRAS* specific primers for *KRAS* codon 12 and 13 (*KRAS*-F, 5′-TTATAAGG CCTGCTGAAAATGACTGAA-3′ and *KRAS*-R, 5′-TGAATTAGCTGTATCGTCAAGGCACT-3′) were based on literature (28) and amplified a 92 bp DNA fragment. Different PCR reagents and enzyme master mixes were used with specific Eprobes during PCR. Eprobes were designed complementary to the reverse strand for mutation detection by the WT probe and to the forward strand for genotyping by the mutant (MT) Eprobe (Table I).

### Highly sensitive mutation detection by high resolution melting analysis

PCR assays were performed using 1.5 *μ*l of 5X LightCycler 480 Genotyping Master, 0.5 *μ*l of DMSO, 2.5 *μ*l of diluted template DNA (2.5 ng/reaction), 0.5 *μ*M of forward primer, 0.1 *μ*M of reverse primer and 0.4 *μ*M Eprobe in a total volume of 10 *μ*l. Amplification reactions and melting curve analysis were from real-time PCR experiments run on a Rotor-Gene Q (Qiagen K.K., Tokyo, Japan) after activation of the hot-start enzyme for 10 min at 95°C, followed by 50 cycles of 15 sec at 95°C, 15 sec at 63°C and 12 sec at 72°C. Amplification signals were detected during the annealing step of each cycle at 63°C, using a SYBR Green I (483 nm) filter. Melting curve analysis and high resolution melting analysis were performed from 40 to 75°C with a temperature increase of 0.5°C/sec. All PCR reactions and melting curve experiments were performed in triplicate.

PCR standard curves were generated by analyzing Ct values acquired from the Rotor-Gene Q instrument with Rotor-Gene Q-Pure Detection (version 2.0.2; Qiagen K.K.). The data was transferred to Microsoft Excel (Microsoft, Redmond, VA, USA) and Ct values were plotted against the template DNA concentrations. Logarithmic trend lines were added, and equations and R-squared values obtained by using Microsoft Excel are shown in the graphs. Microsoft Excel provides slopes as log10(x) values, which were transformed into ln(x) by multiplying the slope value by 2.303 [ln(x) = 2.303 × log10(x)]. The PCR efficiency to evaluate the competing effect is based on seven different concentrations of WT plasmid DNA templates. The slope values were used to calculate PCR efficiencies with a Thermo Fisher online web tool at: http://www.thermoscientificbio.com/webtools/qpcref-ficiency/. Amplification, melting and derivative melting curves were obtained with the Rotor-Gene Q instruments as indicated above, and the data points were transferred to Microsoft Excel for plotting.

### Mutation genotyping by melting curve analysis

Amplification reactions using AmpliTaq Gold were set up in 96-well plates using 5 *μ*l template DNA (50 ng/reaction), 12.5 *μ*l of AmpliTaq Gold PCR Master Mix, 0.1 *μ*M of forward primer, 0.5 *μ*M of reverse primer, and 0.2 *μ*M Eprobe in a total volume of 25 *μ*l. Real-time PCR experiments were run on a Rotor-Gene Q or LightCycler 480 (Roche Diagnostics, Mannheim, Germany) after activation of the hot-start enzyme for 10 min at 95°C, followed by 50 cycles of 15 sec at 95°C, 15 sec at 60°C, and 12 sec at 72°C. Amplification signals were detected during the annealing step of each cycle at 60°C, using a SYBR-Green I (Rotor-Gene Q, 483 nm; LightCycler 480, 483–533 nm) filter. For melting curve analysis, the PCR was followed by heating the reaction mixture to 95°C for 15 sec, cooling to 37°C, holding at 37°C for 7 min, and then slowly heating again to 95°C at a ramp rate of 2.2°C/sec with continuous fluorescence acquisition at the indicated wavelengths. Samples were analyzed in triplicate. Genotyping was performed by the ‘Melting Curve Genotyping’ mode of LightCycler 480 software (version 1.5.1.62; Roche Diagnostics). After setting of the parameters (temperature range, 55–70°C; score threshold, 0.70; and resolution threshold, 0.20), ‘auto grouping’ was carried out for the mutation genotyping by comparison with standard samples for each mutation ratio.

Amplification, melting and derivative melting curves were obtained from the LightCycler 480 instrument as indicated above, and the data points were transferred to Microsoft Excel for plotting.

### Clinical samples

Tumor samples were obtained from patients with CRC who were surgically treated at Gunma University Hospital (Gunma, Japan) between 2002 and 2006. All the samples were immediately frozen after surgical resection and stored at −80°C until DNA extraction. Tumor samples were also preserved in a formalin-fixed paraffin-embedded (FFPE) form. Previous examination with a microscope confirmed that each FFPE tissue sample contained a sufficient number of tumor cells for analysis. In the present study, 92 frozen and 35 FFPE tissue samples were available for gene analysis. An institutional approval and an informed consent from all the patients were obtained in writing.

### DNA extraction

DNA was extracted from a 3- to 5-mm cube of frozen tissues that were collected using a DNA Mini kit (Qiagen K.K.) according to the manufacturer’s instructions. For FFPE samples, two to three 5-*μ*m thick sections were sliced from each block with the maximum number of tumor-rich areas. To suppress normal tissue contamination, the tumor area of the section was macro dissected. DNA was extracted using a QIAamp DNA FFPE tissue kit (Qiagen K.K.) according to the manufacturer’s instructions. After extraction, all the DNA templates were diluted in TE buffer to 10 ng/*μ*l and stored at −20°C. Stored templates were diluted to 1 ng/*μ*l with H_2_O just before Eprobe-PCR with the WT Eprobe assay.

### Sanger sequencing

Mutation screening in clinical samples for *KRAS* codon 12 and 13 was performed using PCR conditions and direct sequencing following previously described protocols (25). The PCR reactions were performed in a final volume of 25 *μ*l containing GeneAmp 10X PCR Gold buffer (Life Technologies, Carlsbad, CA, USA), 1.5 mM of MgCl_2_ solution, 200 *μ*M of dNTPs, 500 nM of each primer (forward primer, 5′-TGAAGTACAGTTCATTACGATACACG-3′ and reverse primer, 5′-GGAAAGTAAAGTTCCCATATTAATGGT-3′), 1 unit of AmpliTaq Gold DNA polymerase (Life Technologies), and 20 ng of genomic DNA. Thermal cycling conditions included preincubation at 94°C for 5 min, followed by 35 cycles at 94°C for 15 sec, 60°C for 30 sec, 72°C for 1 min and extension at 72°C for 5 min. The PCR products were purified using the QIAquick PCR purification kit (Qiagen K.K.) and processed for DNA sequencing reaction using ABI PRISM BigDye Terminator version 3.1 (Applied Biosystems) with a forward primer. Sequence data were generated using the ABI PRISM 3100 DNA Analyzer (Applied Biosystems).

### Next-generation sequencing

Target sequencing libraries for Illumina HiSeq sequencing were prepared using an Illumina TruSeq DNA Library Prep kit (Illumina, San Diego, CA, USA) with multiplexed sample barcoding and amplicon tagging following the manufacturer’s instructions. The Illumina sequencing libraries were run on the Illumina HiSeq 2000 utilizing a 100 bp single end sequencing read protocol.

FASTQ files generated from either the Illumina HiSeq sequencing runs were trimmed with the adapter sequence from sample data with FASTX-toolkit (v0.0.13.2). The trimmed data were mapped to references with the Burrows-Wheeler aligner (v0.6.2-r126). SAM tools (v0.1.18) were utilized to convert the aligned sequence data from a sequence alignment/map (SAM) to a binary sequence alignment (BAM) format. The pileup files from BAM files were created with SAM tools. Single nucleotide polymorphism (SNP) was examined using VarScan2 (v2.3.5jar). SNPs were characterized as being significantly different from the reference sequence if the variant to reference base frequency was >1%.

## Results

### Working principle of mutation enrichment by competitive, allele-specific Eprobe-mediated real-time PCR

**Figure 1:**
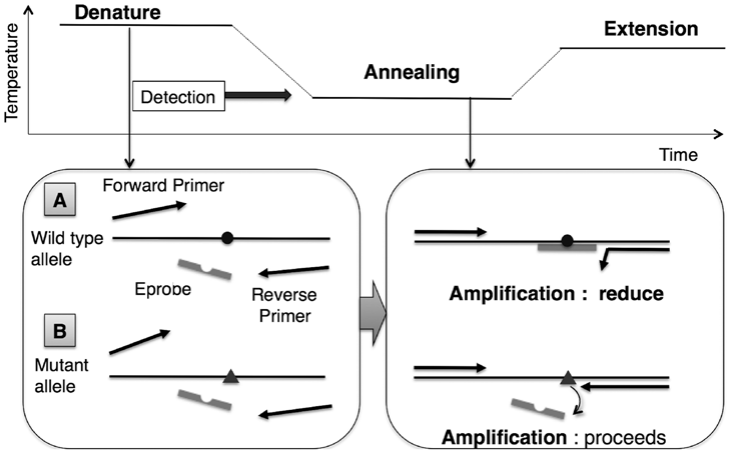
Mutant allele enrichment by Eprobe-PCR using primer competition. (A) Reduction of amplification of the wild-type (WT) template. The Eprobe is designed to match the WT allele sequence (WT Eprobe). WT Eprobe has greater stability to WT template than the reverse primer and reduces its amplification by competing with the reverse primer, resulting in limited amplification of the WT gene. (B) WT Eprobe has less stability to mutant type (MT) template and does not reduce its amplification. (A and B) These combinations enrich the reaction in MT amplicons.

Eprobe-mediated PCR is able to detect the targeted mutation via enrichment of the mutant amplicon by a simple design (Fig. 1). During the reaction, the WT Eprobe binds the WT template with high affinity in the annealing and extension steps, thus preventing generation of the PCR amplicon. In contrast, a mismatch base pair between the WT Eprobe and the mutant template reduces hybridization, resulting in enrichment of the mutant amplicon. The WT Eprobe is designed complementary to the reverse strand for mutation detection (Table I). Standard PCR protocols are used with the exception of an asymmetric primer ratio (*KRAS*-F:*KRAS*-R=5:1) to improve Eprobe binding to the forward strand. The annealing temperature was set to 63°C, which is slightly lower than the Tm value of the full-complementary match between the WT Eprobe and WT template. Taq DNA polymerase lacking 5′-3′ exonuclease activity in LightCycler 480 Genotyping Master is utilized to avoid degradation of WT Eprobe, which binds the WT template during the extension reaction.

### Confirmation of Eprobe-mediated competitive real-time PCR

**Figure 2:**
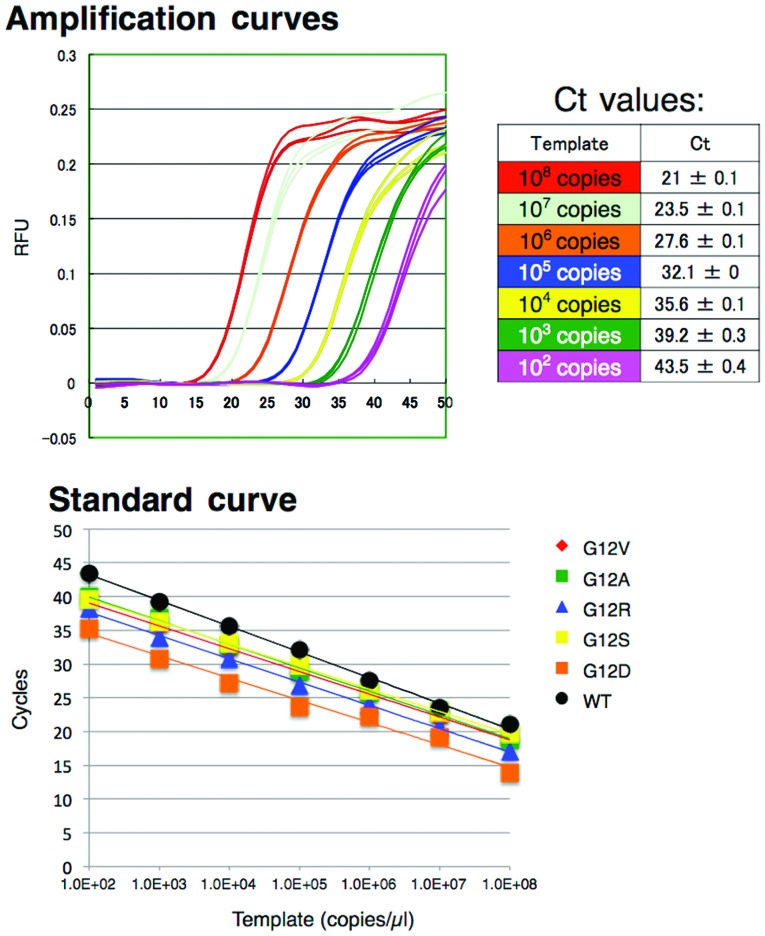
Amplification analysis and PCR efficiency plots for WT plasmid DNA (Ct values plotted against logarithm of plasmid DNA copies/reaction). WT, wild-type.

We tested a serial dilution range from 10^2^ to 10^8^ copies of *KRAS* WT and MT plasmid DNA (Fig. 2). Each dilution was assayed in triplicate. The WT of Eprobe detected amplification of the WT template in real-time and demonstrated a good linear range for detection in the dilution series. Standard curves of triplicate Ct values for each dilution resulted in an 83% amplification efficiency, as calculated from the slope (Fig. 2 and Table II). In contrast, G12C and G13D plasmid DNA templates failed to show amplification curves; however, a mismatch peak was detected on the melting curve analysis (data not shown). These results suggest that the amplifications were successful, but the lower affinity hybridization of the WT Eprobe to the mutant DNA limits the amplification signal. Other MT plasmid templates exhibit good amplification curves and a linear detection range in the dilution series with efficiency values of 93–100% (Table II); thus demonstrating that the competitive effect is inefficient for the MT template, yet present between the WT Eprobe and *KRAS*-R.

### Melting curve analysis and high resolution melting (HRM) analysis by competition with WT Eprobe

We evaluated the ability of *KRAS* low-level mutation detection using 25,000 copies/reaction of plasmid DNA and 2.5 ng/reaction of human genomic DNA. Samples of each mutation ratio were prepared by diluting mutant plasmid DNA with WT plasmid DNA, and then analyzing mutant detection by real-time PCR. Melting curve analysis and high resolution melting (HRM) analysis provided Tm values for the indicated genotypes. The Tm of *KRAS* WT (64.2°C) was higher than the Tm values of all seven *KRAS* mutant types (MTs) in codon 12 and 13 (57.3–54.5°C). For each mutant, a >1% mutation ratio exhibited peaks in the lower region of the Tm values; however, it was difficult to determine values <1% since the curve shapes failed to show distinct peaks.

**Figure 3:**
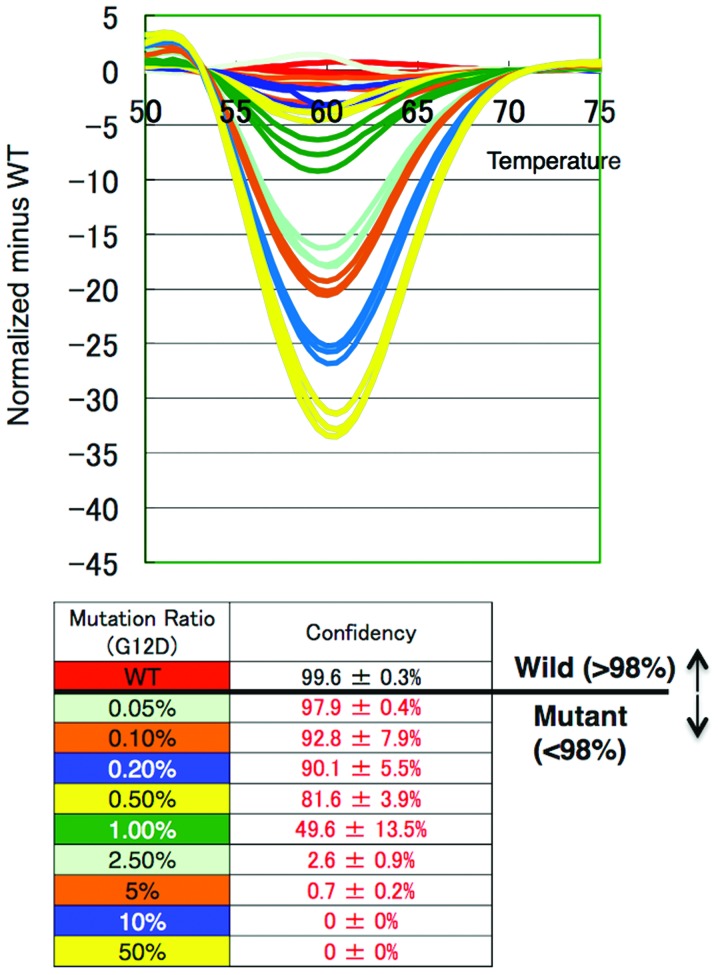
High resolution melting curve analysis and differential plot of each G12D mutation ratio, 0, 0.05, 0.1, 0.2, 0.5, 1.0, 2.5, 5.0, 10 and 50%. Each color represents three replicates. The threshold of *KRAS* mutation detection was set at 98%. *KRAS*, Kirsten rat sarcoma viral oncogene homolog.

On HRM analysis, we detected small changes of curves at <1% mutation ratio by comparing them to the standard curve derived from the WT amplicon (Fig. 3). We evaluated the numerical value from the confidence scores of each sample assigned by the comparison of the melting curves with the WT template. The threshold of *KRAS* mutation detection was set at 98%. Using plasmid DNA, we achieved mutation detection as low as a 0.05–0.1% ratio for each genotype (Table III), yet the human genomic DNA showed an amplification efficiency lower than that of the plasmid DNA. Notably, Eprobe-PCR achieved 1–2.5% mutation detection for each genotype using 2.5 ng of genomic DNA (Table III). These results indicate that Eprobe-PCR with WT Eprobe can detect *KRAS* mutations with high sensitivity even when present at low levels.

### Melting curve analysis by Eprobe for mutation genotyping

**Figure 4:**
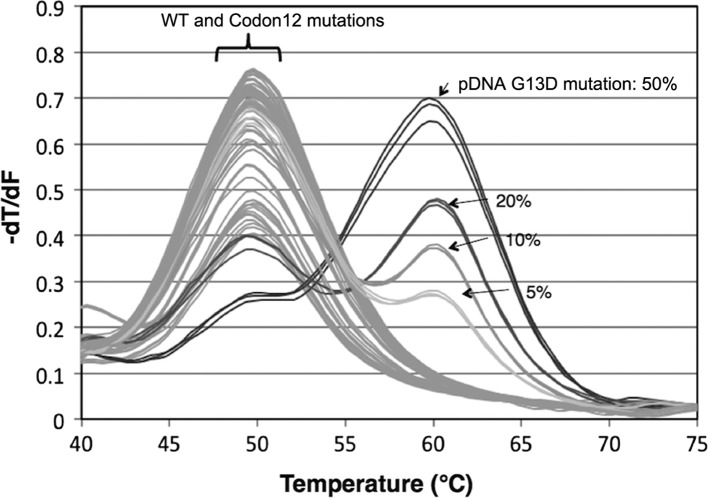
Melting curve of each WT and mutant template using G13D mutation type of Eprobe. Full complementary primer-DNA binding yields peaks in the higher annealing temperature region (Tm=60°C). In comparison, reactions containing other mutants or WT template show mismatch peaks with lower Tm values (50°C). WT, wild-type.

We tested mutation genotyping by MT Eprobes as presented in Table I. The lengths of Eprobes were shorter than that of the WT Eprobe for the low-level mutation detection to avoid competition between the primers and Eprobes. PCR reactions using AmpliTaq Gold Master Mix were run on LightCycler 480 to generate a melting genotyping for each MT template (Fig. 4). Some combinations, such as G12R mutation and G12A MT Eprobe, exhibited a slightly higher Tm value than other mutations and the WT template on melting curve analysis. To confirm this, we used a positive control for each mutation genotype and the highest Tm value to identify the genotype. Importantly, Eprobe-based PCR yielded a genotyping sensitivity as low as a 5 to 10% mutation ratio for each mutant.

### Assay of clinical samples

**Figure 5:**
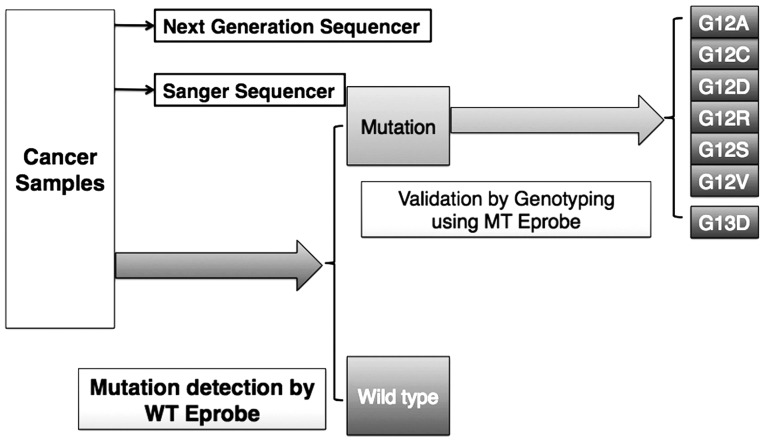
Scheme of mutation detection and genotyping on clinical sample analysis. The WT Eprobe detects the presence of mutations in the *KRAS* locus. The MT Eprobes validate each mutation genotype. WT, wild-type; *KRAS*, Kirsten rat sarcoma viral oncogene homolog; MT, mutant type.

Eprobe-based PCR with WT Eprobe only requires a small amount of DNA sample to *KRAS* mutations; this may then be followed with MT Eprobe PCR to both verify these results as well as genotype the mutation (Fig. 5). To demonstrate the feasibility of Eprobe-based PCR in a clinical setting, we performed the assay on DNA extracted from 92 frozen and 35 FFPE CRC tissue samples. To determine the mutation status with the WT Eprobe, 2.5 ng of genomic DNA from each clinical sample was used per reaction. In the DNA extracted from the frozen tissue, Eprobe mediated PCR by WT Eprobe detected 33/92 mutated samples (36%), whereas Sanger sequencing identified mutation in 20 samples (22%) that were all detected by the Eprobe method (Table IV). These results demonstrate that the Eprobe-PCR method is sufficient to detect *KRAS* mutations using a small amount of clinical sample. Genotyping results with the MT Eprobe identified 20 mutant DNA samples also detected by Sanger sequencing, and 13 that went undetected (Table IV).

We performed a next-generation sequencing (NGS) on these 13 genomic DNA samples that gave differing results with the Eprobe-PCR and Sanger sequencing methods and one that yielded an unclear result with Sanger sequencing (Table V). NGS analyses showed that the 13 DNA samples contained *KRAS* mutations with a 1–10% mutation ratio and were consistent with the Eprobe-PCR results. Furthermore, of the 35 DNA samples extracted from the FFPE tissue, 10 (29%) were positive for *KRAS* mutations that were consistent with the results from the Sanger sequencing (Table VI). These findings demonstrate that Eprobe-PCR with the WT Eprobe allows for the detection of *KRAS* mutations with high sensitivity, consistent to that observed with NGS and more efficient than Sanger sequencing.

## Discussion

In the present study, we have presented a method to detect and genotype somatic *KRAS* mutations in CRC samples using Eprobe-mediated PCR. Eprobes can be used directly for amplification analysis and melting curve analysis for mutation detection and genotyping, respectively. It can also be used as a competitive probe to enrich for mutant amplicon.

The guidelines detailing the design of Eprobes to enrich for mutations via competition-mediated PCR are simple and have been previously published (17). Our group designed and optimized the Eprobes for the present study using the tool ‘ECHO/DNA Thermodynamics’ (18). The WT Eprobe overlaps with the reverse primer at two bases, causing competition during the annealing and extension steps. An annealing temperature slightly lower than the Tm value of the WT Eprobe/template combination (63 vs. 64.2°C) yields a good competition effect. In addition, it is essential to use Taq DNA polymerase that lacks 5′-3′ exonuclease activity, since this activity is able to digest the WT Eprobe during the extension reaction. These optimization techniques effectively prevent amplification of the WT template and lead to enrichment of the mutant amplicons. The Eprobe method exhibits a lower competition effect than PNA and LNA (19–27); however, it also works as a detection probe. The relatively low efficiency value of the WT amplification (83% compared to 93–100%, Table II) allows the WT Eprobe to be used as both a detection probe and a competition probe. This competitive effect enables the detection of mutations present at low ratios (0.05–0.1% mutant in WT background). The results demonstrated the detection of the target sequence by fluorescence and primer competition to reduce WT template amplification with one WT Eprobe. Furthermore, this system simplifies the process to detect *KRAS* mutations in codons 12 and 13, and reduces costs since it only requires a standard primer set, PCR reaction mix and an Eprobe.

To assess Eprobe-mediated PCR performance in a clinical setting, we assayed 92 samples extracted from frozen CRC tissues and evaluated the accuracy of the assay in comparison with the Sanger sequencing. Significantly, out of 92 samples, our WT Eprobe detected the presence of mutations in 33 samples (36%), including 20 (27%) identified as mutants by the Sanger sequencing, and 13 samples (14%) not detected by Sanger but confirmed by NGS. We evaluated this discrepancy in *KRAS* mutation genotyping by Eprobe-mediated PCR with MT Eprobes and NGS (Table V). Eprobes are designed to not overlap with primers and have Tm values below the annealing temperatures of the PCR primers. The MT Eprobes accurately genotyped each of the 33 samples identified as mutants by the WT Eprobe. This demonstrates that Eprobe-mediated PCR is able to detect even a small amount of mutated *KRAS* DNA, which the conventional sequencing method cannot. Sanger sequencing is the gold standard for identification of *KRAS* mutations in codons 12 and 13, yet it has a detection threshold of at least 20% (29). Thus, in a clinical setting, Eprobe-mediated PCR may provide more accurate information for determining which anti-EGFR mAb to use for the CRC patients. NGS may become the primary method to detect somatic mutations in order to ensure the necessary sensitivity and accuracy required by clinical standards. Our Eprobe-mediated PCR method is independent of the NGS method, and offers a more time and cost-effective method with equivalent sensitivity and accuracy; and thus may be a suitable alternative to NGS or a second method to confirm data acquired by NGS.

We also assayed clinical samples from FFPE tissue and found no discrepancy between the results from Eprobe-mediated PCR and Sanger sequencing (Table VI). In general, most clinical samples are fixed and preserved for pathological assessment in hospitals or institutes. However, the DNA extracted from the FFPE tissues is fragmented and chemically modified, making it difficult to use this DNA in molecular studies. The target base length is important for successful PCR when assaying DNA isolated from FFPE samples (32). As the Eprobe and primers in the present study are designed to generate a 92 bp amplicon, the PCR reaction is more likely to be successful even with short fragmented DNA obtained from FFPE tissues. Nevertheless, further investigation with larger sample sizes is required to verify the usefulness of Eprobe-mediated PCR in DNA isolated from FFPE samples.

Conventional real-time PCR-based *KRAS* mutation detection kits have a detection sensitivity of 95–99% and a specificity of 100% (30). Our Eprobe-PCR method satisfies these requirements as it detects mutated DNA (via the WT Eprobe) present at 0.05–0.1% in plasmid DNA and 1–2.5% in human genomic DNA as determined by HRM analysis. This raises the specificity to 100% for detecting mutations using these templates.

The FDA recommends that CRC patient tumor biopsies be assessed for *KRAS* mutation status prior to treatment with anti-EGFR mAbs. Our method has the potential to quickly provide necessary and sufficient information to physicians. It is also likely to reduce the cost of gene sequencing not only for *KRAS*, but also other common oncogenic mutations, including those *BRAF, PIK3CA* and *PTEN* (31).

Eprobe-mediated PCR is superior to other methods, such as Scorpion, ARMS and TaqMelt, in terms of the amount of DNA required. Notably, only 2.5 ng of template DNA samples is needed for detection by Eprobe-PCR, compared to ~100 ng/*μ*l for other methods (11–13). The need for only a small amount of template is beneficial since DNA extraction is laborious and time-consuming. Furthermore, excess template can be used for future analyses with other methods; this is particularly useful in cases where only a small amount of tissue is obtained.

In conclusion, we have developed a novel detection system capable of detecting *KRAS* mutations with high sensitivity using Eprobe-mediated PCR and compared its performance to Sanger sequencing and NGS. The simplicity and high sensitivity of Eprobe PCR may be suitable in a clinical setting that requires swift and accurate *KRAS* gene mutation detection.

## Figures and Tables

**Table I:** Sequences of Eprobe for *KRAS* mutation detection and genotyping.

Eprobe name	Genotype	Length (bases)	Sequence
Rkw19d16	Wild-type	19	5′-CTCzTGCCTAC**GCCACC**AG-3′
K12GCTe3	G12A	14	5′-AGCT**GCTGGC**GzAG-3′
K12TGTe3	G12C	14	5′-AGCT**TGTGGC**GzAG-3′
K12GATe3	G12D	14	5′-AGCT**GATGGC**GzAG-3′
K12CGTe3	G12R	14	5′-AGCT**CGTGGC**GzAG-3′
K12AGTe3	G12S	14	5′-AGCT**AGTGGC**GzAG-3′
K12GTTe3	G12V	14	5′-AGCT**GTTGGC**GzAG-3′
K13GACe3	G13D	14	5′-AGCT**GGTGAC**GzAG-3′

Z, Dye labeling position; underlined, mutation position; bold, *KRAS* codon 12 and 13 region. *KRAS*, Kirsten rat sarcoma viral oncogene homolog.

**Table II:** Efficiency for each mutation type.

Genotype	Sequence (12)	Sequence (13)	Efficiency (%)
**WT**	**GGT**	**GGC**	**83.0**
**G12S**	**AGT**		99.6
**G12C**	**TGT**		ND^a^
**G12R**	**CGT**		94.7
**G12D**	**GAT**		101.3
**G12V**	**GTT**		97.6
**G12A**	**GCT**		93.1
**G13D**		**GAC**	ND^a^

a Not determined due to low affinity to target mutant sequence.

**Table III:** *KRAS* mutation detection.

Mutation	Codon 12	Codon 13	T_m_ (Obs) (°C)	Mutation ratio(pDNA) (%)	Mutation ratio (gDNA) (%)
Wild	GGT	GGC	64.2		
G12A	GCT		54.8	0.05	1.0
G12C	TGT		57.0	0.05	1.0
G12D	GAT		55.5	0.05	1.0
G12R	CGT		57.3	0.05	1.0
G12S	AGT		57.2	0.05	1.0
G12V	GTT		56.2	0.05	2.5
G13D		GAC	54.5	0.1	1.0

Observed, Obs; plasmid DNA, pDNA; genomic DNA, gDNA. *KRAS*, Kirsten rat sarcoma viral oncogene homolog.

**Table IV:** Summary of *KRAS* mutation detection on frozen clinical sample assay.

	Sanger Sequencing (SS)	Eprobe-PCR
No. of samples	92	92
Wild-type status	71	59
Mutated	20	33^a^ (20 +13)
Unclear	1	0

a The 33 cases were 20 mutated cases by SS + 13 mutated cases by Eprobe-PCR but not by SS. *KRAS*, Kirsten rat sarcoma viral oncogene homolog.

**Table V:** Discrepancy of the results for each method.

Sample name	Sanger sequencing	Eprobe mediated PCR		Next-generation sequencing	
		*Mutation detection (WT Eprobe)*	*Genotyping (MT Eprobe)*	*Genotype*	*Mutation ratio (%)*
62	WT	MT	G12V	G12V	7.4
202	WT	MT	G12V	G12V	3.3
207	WT	MT	G12C	G12C	1.7
212	WT	MT	G12V	G12V	2.7
215	WT	MT	G12V	G12V	3.3
217	WT	MT	G12C	G12C	1.4
224	WT	MT	G12D	G12D	6.7
227	WT	MT	G13D	G13D	6.5
230	WT	MT	G12V	G12V	3.0
244D	WT	MT	G13D	G13D	9.5
247T	WT	MT	G13D	G13D	8.9
305	WT	MT	G13D	G13D	6.3
321	Unclear	MT	G13D	G13D	1.1

WT, wild-type; MT, mutant type.

**Table VI:** Results of FFPE samples.

	Eprobe-PCR	Sanger Sequencing (SS)
No. of samples	35	35
Wild-type status	25	25
Mutated	10	10
Unclear	0	0

FFPE, formalin-fixed paraffin-embedded (FFPE).
